# Specialized care improves outcomes for patients with cirrhosis who require general surgical operations

**DOI:** 10.1371/journal.pone.0223454

**Published:** 2019-10-16

**Authors:** Joshua K. Kays, Daniel P. Milgrom, James R. Butler, Tiffany W. Liang, Nakul P. Valsangkar, Brandon Wojcik, C. Corbin Frye, Mary A. Maluccio, Chandrashekhar A. Kubal, Leonidas G. Koniaris

**Affiliations:** Department of Surgery, Indiana University School of Medicine, Indianapolis, IN, United States of America; Harvard Medical School Teaching Hospital, UNITED STATES

## Abstract

**Background:**

General surgical operations on patients with cirrhosis have historically been associated with high morbidity and mortality rates. This study examines a contemporary series of patients with cirrhosis undergoing general surgical procedures.

**Methods:**

A retrospective evaluation of 358 cirrhotic patients undergoing general surgical operations at a single institution between 2004–2015 was performed. Thirty- and 90-day mortality along with complications and subsequent transplantation rates were examined.

**Results:**

358 cirrhotic patients were identified. The majority were Child-Turcotte-Pugh class (CTP) A (55.9%) followed by class B (32.4%) and class C (11.7%). Mean MELD score differed significantly between the groups (8.7 vs. 12.1 vs. 20.1; p<0.001). The most common operations were herniorrhaphy (29.9%), cholecystectomy (19.3%), and liver resection (14.5%). The majority of cases were performed semi-electively (68.4%), however, within the CTP C patients most cases were performed emergently (73.8%). Thirty and 90-day mortality for all patients were 5% and 6%, respectively. Mortality rates increased from CTP A to CTP C (30 day: 3.0% vs. 5.2% vs. 14.3%; p = 0.01; 90 day: 4.5% vs. 6.9% vs. 16.7%; p = 0.016). Additionally, 30-day mortality (12.8% vs. 2.3%; p<0.001), 90 day mortality (16.0% vs. 3.4%; p<0.001) were higher for emergent compared to elective cases. A total of 13 (3.6%) patients underwent transplantation ≤ 90 days from surgery. No elective cases resulted in an urgent transplantation.

**Conclusion:**

Performing general surgical operations on cirrhotic patients carries a significant morbidity and mortality. This contemporary series from a specialized liver center demonstrates improved outcomes compared to historical series. These data strongly support early referral of cirrhotic patients needing general surgical operation to centers with liver expertise to minimize morbidity and mortality.

## Introduction

According to the National Institute of Diabetes and Digestive and Kidney Disorders, approximately 1 in 400 adults in the United States has cirrhosis [1]. This translates to over 600,000 adult patients with some suggestion that this is likely underestimated [2]. With the epidemic rise of nonalcoholic fatty liver disease as previously under recognized cause of cirrhosis, complications of liver disease and cirrhosis are also expected to increase [2,3]. This will invariably lead to increased complications associated with surgical procedures required within this vulnerable patient population.

Performing abdominal operations on cirrhotic patients is a major challenge with reported mortality rates as high as 80% [4,5]. Many have studied the patient factors that contribute to the increased operative risk associated with cirrhosis. The Child-Turcotte-Pugh (CTP) and Model for End-Stage Liver Disease (MELD) scores are two validated, widely utilized systems to estimate perioperative mortality in patients with cirrhosis [6–8]. The scoring systems, however, focus on patient factors and ignore whether these factors would be blunted by the availability of specialized resources within any given hospital facility.

Volume-outcome relationships have been established for several high-risk operations. Esophageal, pancreatic, sarcoma and bariatric operations, along with coronary artery bypass graft, abdominal aortic aneurysm repair and carotid endarterectomy have all been shown to have lower mortality rates when performed at high volume institutions [9–13]. The difference in mortality rates between high- and low-volume centers for many of these conditions is not necessarily attributed to differences in complications rates, but rather in the ability of a surgical team and hospital system to effectively rescue patients from complications [14].

The aim of this study was to evaluate contemporary rates of perioperative morbidity, mortality, and progression to transplantation within the cirrhotic population undergoing general surgical operations at a high-volume center with expertise in liver disease, management of ascites and the availability of salvage transplant.

## Materials and methods

This study was approved by the Indiana University Institutional Review Board and was carried out in compliance with the IU Standard Operating Procedures for Research Involving Human Subject. All data collection occurred between July 1, 2015 and June 30, 2017. Due to the retrospective nature of the study the IRB waived the requirement to obtained informed consent. All patient data was anonymized at the time of collection and a master reference sheet was kept on a password protected Microsoft Excel file which was on an encrypted USB flash drive kept in the locked office of the corresponding author (LGK).

All patients who underwent general surgery procedures in the context of hepatic decompensation at Indiana University Medical Center (IUMC) between January 2004 and January 2015 were eligible for inclusion and subject to medical records review. Patients undergoing general surgery procedures at IUMC were cross-referenced for a diagnosis of hepatic failure (ICD-9 codes 520–579; ICD9 codes were used for data collection purposes) over the same 10-year period. Patient charts were then individually reviewed for confirmation of a diagnosis of cirrhosis by either pathologic examination of a liver biopsy or intra-operative findings consistent with cirrhosis. This resulted in 358 total patients. A retrospective review of these patient’s medical records was then performed to collect all variables of interest.

Patients were divided by severity of liver disease using Child-Turcotte-Pugh (CTP) classification [6,7]. Urgency of operation was divided into two groups: elective, which included all patients who presented for a planned operation, and emergent, which included all patients who did not present with a planned operation but underwent one during the same hospitalization. Model for End-Stage Liver Disease (MELD) score was calculated as has been previously described [8]. CTP and MELD score were both calculated using data obtained prior to operative intervention. Race was grouped into 4 categories: White, Black, Hispanic, and Asian. Operations were grouped based by organ system. Body mass index (BMI) was calculated by standard means [15]. Major complications were defined by Clavien-Dindo class ≥ 3 [16].

The primary outcome of interest was 30-day mortality of each CTP class. Secondary outcomes included 90-day mortality and progression to transplantation based on CTP class, and 30- and 90-day mortality and progression to transplantation based on urgency of operation. Relative risk was calculated between groups in the standard fashion and reported with 95% confidence intervals (CI) and p values.

All continuous variables are reported as mean with total range. All categorical variables are reported as the measured value with percentage. Continuous variables were compared using two-sided T test or ANOVA where appropriate. When ANOVA revealed a difference Tukey’s post-hoc test was utilized to reveal the difference. Categorical variables were compared using Pearson’s chi-squared test. Statistical analysis was performed using IBM SPSS Statistics software version 24.0) (SPSS, Armonk, NY). All p values are two-sided. Significance was set at a p value <0.05 for all results.

## Results

Patient demographics are shown in Table 1. Mean age was 55.7 (20.4–86.9) years with no significant difference in age between CTP classes. There were slightly more males than females, 55.6% vs. 44.4%, with no difference in distribution between groups. The majority of patients were white, 90.5%. Mean MELD score was significantly different between the 3 groups (p<0.001), increasing from CTP A to CTP C. Most procedures were performed on an elective or semi-elective basis (68.4%). However, in the CTP C group the majority of operations were performed in an emergent setting (73.8%). Table 2 shows a breakdown of the operations performed with herniorrhaphy (29.9%), cholecystectomy (19.3%), and liver operations (14.5%) being the most common.

**Table 1:** Patient demographics (N = 358).

	Overall	CTP A (n = 200)	CTP B (n = 116)	CTP C (n = 42)	P
Age (yrs)	55.7 (20.4–86.9)	56.1 (204–86.9)	55.1 (25.1–84.4)	55.5 (23.0–74.0)	0.794
Sex					
- Male	199 (55.6%	103 (51.5%)	70 (60.3%)	26 (61.9%)	0.213
- Female	159 (44.4%)	97 (48.5%)	46 (39.7%)	16 (38.1%)	
Race					
- White	324 (90.5%)	181 (90.5%)	107 (92.2%)	36 (85.7%)	0.665
- Black	27 (7.5%)	15 (7.5%)	8 (6.9%)	4 (9.5%)	
- Hispanic	2 (0.6%)	1 (0.5%)	0 (0%)	1 (2.4%)	
- Asian	5 (1.4%)	3 (1.5%)	1 (0.9%)	1 (2.4%)	
BMI (kg/m^2^)	29.9 (14.4–62.2)	31.0 (16.0–62.2)	28.3 (14.4–48.9)	29.9 (20.0–57.5)	0.011
MELD	11.2 (6.4–36.4)	8.7 (6.4–34.8)	12.1 (6.4–31.8)	20.1 (9.6–36.4)	<0.001
LOS (days)	8.6 (0–155)	6.3 (0–115)	9.0 (0–58)	18.6 (0–155)	<0.001
Op Time (min)	160.4 (22–618)	175.1 (24–598)	154.3 (25–618)	18.6 (0–155)	<0.001
Urgency					
- Emergent	113 (31.6%)	29 (14.5%)	53 (45.7)	31 (73.8%)	<0.001
- Elective	245 (68.4%)	171 (85.5%)	63 (54.3%)	11 (26.2%)	

Abbreviations: CTP–Child-Turcotte-Pugh; yrs–years; BMI–body mass index; MELD–Modified End Stage Liver Disease score; LOS–length of stay; Op time–operative time

**Table 2:** Operations performed.

	Overall (%)	CTP A	CTP B	CTP C
Appendectomy	1 (0.3)	0	1	0
Cholecystectomy	69 (19.3)	44	22	3
Herniorrhaphy^a^	107 (29.9)	45	37	25
Gastric	12 (3.4)	6	5	1
Small Intestine	14 (3.9)	10	3	1
Colon	20 (5.6)	11	8	1
Pancreas	29 (8.1)	17	10	2
Liver	52 (14.5)	45	7	0
Spleen	3 (0.8)	1	1	1
Exploratory Laparotomy	19 (5.3)	8	8	3
Lymphadenectomy	13 (3.6)	3	7	3
Adrenal	3 (0.8)	2	1	0
Other	16 (4.5)	8	6	2
Total	358 (100)	200 (55.9)	116 (32.4)	42 (11.7)

Abbreviations: CTP- Child-Turcotte-Pugh

a–includes all hernias whether inguinal, umbilical or ventral

To characterize the effects of severity of liver disease on survival, 30-day and 90-day mortality were compared between CTP classes. CTP A patients had lower 30-day and 90-day mortality than CTP B patients which in turn had lower rates than CTP C patients (Table 3). CTP B patients were more likely to undergo transplantation within 90 days than CTP A patients (RR = 12.1; CI = 1.5–96.9) but demonstrated no increased risk for 30-day or 90-day mortality (Table 4). CTP C patients are at increased risk of 30-day mortality (RR = 4.8; CI = 1.6–14.0), 90-day mortality (RR = 4.8; CI = 1.9–12.0) and rate of undergoing transplantation within 90 days (RR = 23.8;CI = 2.9–198.6) versus CTP A patients (Table 4). CTP C patients demonstrated increased risk of 90-day mortality (RR = 2.8; CI = 1.1–6.9) but no increased risk of 30-day mortality or transplantation within 90 days than CTP B patients (Table 4).

**Table 3:** CTP mortality and 90 day transplant rates.

	Overall (%)	CTP A	CTP B	CTP C	P value
30 day mortality					
- Yes	18 (5.0)	6 (3.0)	6 (5.2)	6 (14.3)	0.010
-No	340 (95.0)	194 (97.0)	110 (94.8)	36 (85.7)	
90 day mortality					
- Yes	24 (6.7)	9 (4.5)	8 (6.9)	7 (16.7)	0.016
- No	334 (93.3)	191 (95.5)	108 (93.1)	35 (83.3)	
Transplant ≤ 90 days					
- Yes	13 (3.6)	1 (0.5)	7 (6.0)	5 (11.9)	<0.001
-No	345 (96.4)	199 (99.5)	109 (94.0)	37 (88.1)	

Abbreviations: CTP–Child-Turcotte-Pugh

**Table 4:** Relative risk.

**CTP A vs. CTP B**			
	RR	95% CI	P value
30-day mortality	1.7	0.6–5.2	0.33
90-day mortality	1.7	0.7–4.5	0.26
Transplant ≤ 90 days	12.1	1.5–96.9	0.02
**CTP A vs. CTP C**			
30-day mortality	4.8	1.6–14.0	0.0047
90-day mortality	4.8	1.9–12.0	0.0009
Transplant ≤ 90 days	23.8	2.9–198.6	0.0034
**CTP B vs. CTP C**			
30-day mortality	2.8	0.9–8.1	0.0640
90-day mortality	2.8	1.1–6.9	0.0294
Transplant ≤ 90 days	2.0	0.7–5.9	0.2227
**Emergent vs. Elective Operations**			
30-day mortality	4.3	1.7–11.3	0.0026
90-day mortality	3.6	1.6–8.0	0.0016
Transplant ≤ 90 days	114.8	28.9–456.3	<0.0001

Abbreviations: CTP- Child-Turcotte-Pugh

All patients, regardless of CTP class, undergoing an emergent operation had increased 30-day mortality (10.6% vs. 2.4%; p = 0.001), 90-day mortality (13.3% vs. 3.7%;p = 0.001), and increased rate of transplantation within 90 days (9.7% vs. 0.8%;p<0.001) compared to patients undergoing elective operations (Table 5). Relative risk for 30-day mortality, 90-day mortality, and transplantation within 90 days were 4.3 (CI = 1.7–11.3), 3.6 (CI = 1.6–8.0), and 114.8 (CI = 28.9–456.3), respectively when comparing emergent to elective procedures (Table 5).

**Table 5:** Emergent vs. Elective mortality and 90 day transplant rates.

	Emergent (%)	Elective (%)	P value
30 day mortality			
- Yes	12 (10.6)	6 (2.4)	0.001
- No	101 (89.4)	239 (97.6)	
90 day mortality			
- Yes	15 (13.3)	9 (3.7)	0.001
- No	98 (36.7)	236 (96.3)	
Transplant ≤ 90 days			
- Yes	11 (9.7%)	2 (0.8%)	<0.001
- No	102 (90.3)	243 (99.2)	

Table 6 shows a breakdown of post-operative complications. A total of 75 (21.0%) patients had at least one post-operative complication, of which 52 (14.5%) had a major complication. There was no difference in overall or major complication rates when comparing CTP class or elective vs. emergent cases. Of the 52 patients experiencing a major complication 9 (17.3%) died within 30 days, 14 (26.9%) within 90 days, and 1 (1.9%) patient went on to transplantation within 90 days. Ability to rescue was significantly different among CTP classes. Progression to death within 30 days and 90 days was significantly higher as CTP class increased. This held true for elective vs. emergent cases as well with patients who experienced a complication after emergent surgery progressing to death within both 30 and 90 days more frequently than similar patients who underwent elective surgery.

**Table 6:** Post-operative complications and rescue rates.

	Overall (%)^b^	CTP A	CTP B	CTP C	P	Elective	Emergent	P
Any complication	75 (21.0)	41 (20.6)	26 (22.4)	8 (19.00)	0.88	52 (21.3)	23 (20.4)	0.84
Superficial Incisional Surgical Site Infection	19 (5.3)	14 (7.0)	3 (2.6)	2 (4.8)	0.24	16 (6.5)	3 (2.7)	0.13
Deep Incisional Surgical Site Infection	4 (1.1)	2 (1.0)	2 (1.7)	0 (0)	0.64	3 (1.2)	1 (0.9)	0.78
Wound Disruption/Dehiscence	2 (0.6)	2 (1.0)	0 (0)	0 (0)	0.45	1 (0.4)	1 (0.9)	0.57
Pneumonia	14 (3.9)	5 (2.5)	7 (6.0)	2 (4.8)	0.28	8 (3.3)	6 (5.3)	0.35
Progressive Renal Insufficiency	4 (1.1)	0 (0)	4 (3.4)	0 (0)	0.02	1 (0.4)	3 (2.7)	0.06
Urinary Tract Infection	10 (2.8)	7 (3.5)	2 (1.7)	1 (2.4)	0.64	9 (3.7)	1 (0.9)	0.14
Unplanned Intubation	25 (7.0)	11 (5.5)	11 (9.5)	3 (7.1)	0.41	15 (6.1)	10 (8.8)	0.35
Major Complications	52 (14.5)	23 (11.5)	22 (19.0)	7 (16.7)	0.18	31 (12.7)	21 (18.6)	0.14
Deep Organ Space Surgical Site Infection	15 (4.2)	7 (3.5)	7 (6.0)	1 (2.4)	0.46	9 (3.7)	6 (5.3)	0.47
Pulmonary Embolism	0	0	0	0	N/A	0	0	N/A
Mechanical Ventilation > 48 hours	27 (7.5)	12 (6.0)	12 (10.3)	3 (7.1)	0.37	15 (6.1)	12 (10.6)	0.13
Unplanned Intubation	25 (7.0)	11 (5.5)	11 (9.5)	3 (7.1)	0.41	15 (6.1)	10 (8.8)	0.35
Sepsis	17 (4.7)	6 (3.0)	10 (8.6)	1 (2.4)	0.06	11 (4.5)	6 (5.3)	0.74
Septic Shock	17 (4.7)	7 (3.5)	7 (6.0)	3 (7.1)	0.44	7 (2.9)	10 (8.8)	0.01
Acute Renal Failure	7 (2.0)	3 (1.5)	2 (1.7)	2 (4.8)	0.38	4 (1.6)	3 (2.7)	0.52
Cerebrovascular Accident	0	0	0	0	N/A	0	0	N/A
Cardiac Arrest requiring CPR	1 (0.3)	0	1 (0.9)	0	0.35	0 (0)	1 (0.9)	0.14
Myocardial Infarction	0	0	0	0	N/A	0	0	N/A
DVT requiring anticoagulation	6 (1.7)	5 (2.5)	1 (0.9)	0 (0)	0.37	6 (2.4)	0 (0)	0.09
Progressed to Death ≤ 30 days	9 (17.3)^a^	2 (8.7)^a^	4 (18.2)^a^	3 (42.9)^a^	0.05	2 (6.5)^a^	7 (33.3)^a^	0.003
Progressed to Death ≤ 90 days	14 (26.9)^a^	4 (17.4)^a^	6 (27.3)^a^	4 (57.1)^a^	0.05	5 (16.1)^a^	9 (42.9)^a^	0.007
Progressed to Transplantation ≤ 90 days	1 (1.9)^a^	0 (0.0)	1 (4.5)	0 (0)	0.35	0 (0)	1 (0.9)	0.14

Abbreviations: CTP–Child-Turcotte-Pugh; DVT–deep venous thrombosis

a–Percentages are based on total major complications

b–the sum of individual complications will not equal total complications because patients may have experienced multiple complications. Patients with multiple complications were counts as one for any complication and major complication overall total.

## Discussion

Patients with liver cirrhosis represent a unique challenge to the general surgeon. Traditionally mortality rates range from 10% to 80% depending on the severity of the liver dysfunction [4,5]. This contemporary series utilized careful preoperative optimization and salvage transplantation in select patients demonstrates significantly lower 30-day and 90-day mortality rates than previous series.

Consistent with previous data, this study suggests the risk of post-operative mortality increases with the severity of liver disease. The current study, however, demonstrates 30-day mortality rates of 3%, 5.2%, and 14.3% and 90-day mortality rates of 4.5%, 6.9%, and 16.7%, for CTP A, B, and C patients, respectively (Table 3) which are significantly lower than the traditionally reported rates of 10%, 30%, and 80% [4,5]. This represents a dramatic improvement in post-operative mortality and attention should be focused on how these results were achieved in this high-risk population.

Recent data has proposed surgical volume to be associated with outcomes for high-risk procedures. Studies on high-risk surgery such as esophageal, pancreatic, bariatric, sarcoma, coronary artery bypass grafting, abdominal aortic aneurysm repair and carotid endarterectomy have shown improved outcomes at high volume facilities [9–13]. The current data represent 358 cirrhotic patients treated over a 10-year period, translating to approximately 3 operations per month, thus making the institution a high volume center. There are, however, 2 major issues with the volume-outcomes relationship standard. The first being that the exact mechanism by which volume improves outcomes has yet to be defined and the second is there is no discrete threshold that defines low- vs. high-volume. Unfortunately, the current study was not designed to answer either of these questions, however, it is reasonable to say that familiarity with a patient population leads to expertise, which subsequently results in improved outcomes.

There is a lack of consensus that high-volume is necessarily the driver of improved outcomes for high-risk operations. Schell et al. reported low-volume hospitals being able to achieve equivalent post-operative complication and perioperative mortality rates after pancreatoduodenectomy provided the expertise and necessary care pathways were present [17]. Joseph et al. took this concept a step further to show that system clinical resources were more influential in operative mortality for pancreatic resection than volume itself [18]. Finks et al. showed that while improved outcomes in high-risk cancer operations could be attributed to higher volume, other factors, such as improved technology, operating room protocols, and perioperative care are responsible for the lower mortality rates associated with other high-risk procedures [19]. Other factor such as nutrition, access, and socioeconomic status have also been associated with outcomes in high-risk operations [20–22]. These non-volume factors likely had an effect on the results of this study. A center of excellence, regardless of the volume of any particular case, has surgeons with expertise in the management of the surgical issue, but also liver disease. Anesthesiology, hepatology, interventional radiology, nephrology, infectious disease, critical care staff, and senior surgical residents with significant experience on hepatopancreatobiliary and liver transplant services also contribute to the institutional expertise. A multidisciplinary approach leads to early recognition and active management of complications and improved outcomes. Therefore, in the management of general surgical procedures in patients with cirrhosis, and particularly in patients with advanced end stage liver disease requiring an urgent or emergent operation, the combination of high-volume, clinical expertise, and vast ancillary support all have significant roles in the dramatically improved outcomes. At the authors institution all of these services are available and collaborate routinely. While the exact breakdown of the different specialists involved in each case is not available for the current study, a multidisciplinary approach was used for each patient with each specialty involvement being tailored to each individual situation.

Post-operative complications are common in patients with cirrhosis [23]. Overall morbidity has been reported as high as 50% with CTP class specific mortality being approximately 40% for A and > 60% for both B and C [24–26]. Overall morbidity in the current study was 21.0%, and the rate of major complications was 14.2% (Table 1). While there was no difference in morbidity rates, the ability to rescue patients after a major complication was significantly different between the CTP classes. Increasing CTP score was associated with a significant increase in 30-day and 90-day mortality after a major complication (Table 6). This is not unexpected as these patients by default are sicker with less physiologic reserve to withstand an additional insult. In keeping with data comparing elective versus emergent cases, patients undergoing emergency surgery with a major post-operative complication had higher 30-day and 90-day mortality rates than patients with similar risk factors undergoing surgery under elective circumstances. The incarcerated umbilical hernia or complicated cholecystitis that drive the need for emergent operations are common conditions where the initial delay in intervention was likely due to concerns over complications related to the liver disease only for the condition to become emergent. The data presented here suggests that it would be of far greater value to perform the operation in the elective setting than to continue with non-operative management due to concerns over postoperative recovery. Despite almost 50% of patients in this study having Child’s B or greater cirrhosis and a higher proportion of CTP C patients needing emergent operations, the likelihood of needing a salvage liver transplant remained low. However, for patients identified as higher risk for fulminant liver failure, having the expertise of a liver transplant team readily available and the components in place for an expeditious evaluation would be a more efficient utilization of hospital based resources for this vulnerable patient population.

While one would anticipate that lower complication rates result in lower mortality rates, this is not always the case. In a study published by Ghaferi et. al, they showed the differences in mortality were not necessarily due to differences in complication rates but rather differences in the ability to rescue patients from complications [14]. Of the 51 patients that experienced a major post-operative complication, the majority were rescued and only 9 (17.6%) died within 30 days and 14 (27.5%) died within 90 days (Table 6). Although it is difficult to explain the unique circumstances that precluded transplant for these 14 patients, our experience as a transplant referral center is that there are some insurmountable comorbid issues (cardiovascular disease, social support, toxicology, or sepsis) that preclude liver transplant under any circumstances and that these same issues impacted the patients who died in this series. The success of transplant in a handful of patients was also likely multi-factorial, but important factors are time to recognition and time to intervention. These factors are often dependent on the hospital expertise, capabilities, and resources available.

Elective abdominal operations have been shown to be associated with a significantly lower mortality than emergent cases [4,27,28]. The results of the current study echo previous findings. Thirty-day and 90-day mortality were lower for elective cases compared to emergent cases (Table 4). Furthermore, emergent procedures carried a significantly increased relative risk for both 30-day (RR = 4.3) and 90-day (3.6) mortality (Table 5). Additionally, the 30-day and 90-day mortality for all elective operations performed in this study were acceptably low at 2.4% and 3.7% (Table 4). This data suggests adopting a more aggressive approach by operating on cirrhotic patients early in an elective manner when possible rather than a watch and wait approach and risking the need to perform an operation in an emergent setting.

The increased morbidity and mortality seen in operating on patients with cirrhosis is most often a direct result of hepatic dysfunction [29]. The only definitive treatment for cirrhosis with correction of the hepatic dysfunction remains liver transplantation. Thirteen patients in this study went on to liver transplantation (LT) within 90 days surgery (Table 3). While there was no evidence in this study that LT was necessary for treatment of acute fulminant hepatic decompensation from the operation, LT does represent a potential rescue therapy if this were to occur. At our facility a patient with acute fulminant hepatic decompensation can undergo all required testing and be listed for transplantation within 24 hours. The biggest factor in delay is typically an inability to recognize failure to rescue and the need for LT. Having transplantation expertise and capabilities on site may result in earlier evaluation and recognition of failure to rescue, which in turn would minimize delays for patients in need of LT.

This study is not without limitations. First, this is a retrospective review and is subject to all limitations as such. Secondly, the underlying cause of the cirrhosis was not included in the analysis. This was due to the lack of concrete documentation of the underlying cause and thus no meaningful analysis could be performed on the impact of the underlying causes. The vast majority of the cohort were caucasian, however, this is not a significant limitation because there is no evidence that cirrhosis effects different races in different manners. Post-operative complications were collected from the institutions NSQIP database, and some liver specific complications such as new onset or worsening ascites or signs of decreased hepatic function such as increased prothrombin/International Normalized Ratio (PT/INR) were not collected. The authors acknowledge the complication rates reported here may increase if additional liver specific complications were collected, however, due to resource limitations this was not feasible at this time. There were 13 patients who underwent LT within the 90-day period. The authors recognize that a timely transplant impacted the potential for ongoing complications and risk of death had they been ineligible for transplant, improving the outcomes in a subgroup of patients with the highest mortality. It was not clear, however, if the preceding operation resulted in the need for early transplant. Additionally the authors acknowledge that type II error may exist in some comparisons due to small numbers. This is unfortunately unavoidable due to the overall low prevalence of operations performed on cirrhotics.

## Conclusion

Patients with cirrhosis represent a challenging patient population for the general surgeon. These patients have physiologic alterations that put them at increased risk for morbidity and mortality after surgery. The data presented here show significantly improved morbidity and mortality rates compared to those that have been traditionally reported. The cause of this improvement is likely multi-factorial, with volume, level of expertise, and clinical support available all playing important roles. If possible these operations should be performed on a planned, elective basis. Early referral to high-volume facilities with liver expertise and strong clinical support is imperative to optimizing outcomes for patients with cirrhosis in need of general surgical operations.

## Conflict of interest

Joshua K. Kays, Daniel P. Milgrom, James R. Butler, Tiffany W. Liang, Nakul P. Valsangkar, Brandon Wojcik, C. Corbin Frye, Mary A. Maluccio, Chandrashekhar A. Kubal, and Leonidas G. Koniaris certify that they have NO affiliations with or involvement in any organization or entity with any financial interest (such as honoraria; educational grants; participation in speakers’ bureaus; membership, employment, consultancies, stock ownership, or other equity interests; and expert testimony or patent-licensing arrangements), or non-financial interest (such as personal or professional relationships, affiliations, knowledge or beliefs) in the subject matter or materials discussed in this manuscript. There was no external funding for this project.

## Supporting information

S1 Dataset (XLSX)
